# Interaction effect of coping self-efficacy and received support in daily life of hematopoietic cell transplant patient-caregiver dyads

**DOI:** 10.1371/journal.pone.0260128

**Published:** 2021-11-17

**Authors:** Aleksandra Kroemeke, Małgorzata Sobczyk-Kruszelnicka

**Affiliations:** 1 Department of Health Psychology, Faculty of Psychology in Warsaw, SWPS University of Social Sciences and Humanities, Warsaw, Poland; 2 Department of Bone Marrow Transplantation and Oncohematology, Maria Skłodowska-Curie National Research Institute of Oncology (MSCNRIO) Gliwice Branch, Gliwice, Poland; University of Bologna, ITALY

## Abstract

**Objectives:**

According to the social cognitive theory, social support and self-efficacy may interact with each other i.e. compete or account jointly for better adaptation. This study examined the nature of the interaction between coping self-efficacy and received social support in daily lives of patient-caregiver dyads after cancer treatment. We tested whether the effect of daily fluctuations in coping self-efficacy and received support on daily affect was synergistic (positive jointed effect), compensatory (positive competing effect), or interference (negative competing effect).

**Design:**

A dyadic daily-diary study conducted for 28 days after hospital discharge following hematopoietic cell transplantation.

**Methods:**

Coping self-efficacy, received support, and positive and negative affect were measured in 200 patient-caregiver dyads. The analysis was based on the actor-partner interdependence moderation model using multilevel structural equation modeling.

**Results:**

Statistically significant effect of interaction between daily coping self-efficacy and received support on negative affect was found, although only in the caregivers. In that group, higher daily received support compensated for lower daily coping self-efficacy but had a negative effect when coping self-efficacy was significantly higher than typical. Also, direct beneficial effects of higher daily coping self-efficacy and received support on caregiver positive affect were found. In the patients, higher daily coping self-efficacy was directly associated with better daily affect.

**Conclusions:**

Diverse effects of daily coping self-efficacy and received social support were found—the interference effect in the caregivers and the main effect of coping self-efficacy in the patients. Higher daily coping self-efficacy and optimal received social support may provide resilience against affect disturbance after cancer treatment.

## Introduction

Adaptation to a chronic disease is a complex process involving numerous factors which fluctuate over time [1]. Undoubtedly, disease-related demands influence both, patients and their caregivers, becoming shared stressors [2]. The role of individual and social resources during that stressful time has been highlighted in the literature [1, 2].

### The role of self-efficacy beliefs in adaptation to cancer

As far as individual resources are concerned, self-efficacy beliefs have often attracted the attention of the researchers. Both, perceived (i.e. the perceived capability to manage one’s functioning) and coping self-efficacy (i.e. the perceived capability to manage external and internal demands) are central components of self-evaluation, which—according to the social cognitive theory—is a crucial mechanism for self-regulation and effective adaptation in stressful circumstances [3, 4]. Previous studies have demonstrated positive associations between self-efficacy beliefs and the recovery process of cancer populations [5–11] or caregiver adaptation to cancer [12, 13]. Furthermore, positive effects of coping self-efficacy were found across partners (i.e. patient to caregiver and caregiver to patient) dealing with prostate cancer [12]. Longitudinally, coping self-efficacy predicted better well-being in women with breast cancer [14] and in hematopoietic cell transplantation survivors at 1 year follow-up [15], as well as well-being in patients after cancer surgery at 5-month follow-up [16].

### Received support and adaptation to cancer

Functional social support has been identified as the main social resource which facilitates adaptation to chronic disease [2]. Conceptually, functional support may be received to bring benefits or may only be perceived to become the available support [2]. In a fluctuating patient-caregiver relationship, the former conceptualization seems more accurate: Actually received support is dynamic and can change on daily basis, while perceived support is believed to be a stable individual disposition [17]. Support in a patient-caregiver relationship is a mutual experience, where both parties give and receive support [2]. Contrary to the findings about self-efficacy, there is no consensus regarding the beneficial effects of received support on well-being in cancer patients and caregivers. Received support was found to have positive effects in cancer populations [18–21] and caregivers [19, 21], while other studies found null effect on patients and caregivers [22, 23], including cross-partner effects [19].

### Interplay between self-efficacy beliefs and received support

One hypothesis about mixed effects of received support on well-being has roots in the social cognitive theory [3]. According to this approach, individual and social resources interact with each other and successful adaptation to a stressful encounter is the product of an interplay between them [24]. Previous research found that coping self-efficacy and support received from a caregiver accounted jointly for the variance of patient well-being after surgery [16]. Thus, effective adaptation to cancer may be conditioned by sufficiently high levels of both resources (synergistic hypothesis). However, better outcomes may also be observed when only one of the resources is high, suggesting either a compensatory or an interference mechanism of their interaction. Compensation is a product of positive resource interaction and identifies those resources which are sufficient for better adaptation [25]. For example, social support was found to compensate for efficacy deficits in cardiac patients [25, 26] and those with rheumatoid arthritis [27]: Negative association between social support and distress was found in low self-efficacy patients, whereas no or weak association was observed in patients with high self-efficacy. In contrast, interference is a result of negative resource interaction identifying sufficient and optimal resources for adaptation [25]. The interference effect emerged in older adults with chronic diseases [28]: Received support increased autonomy in low self-efficacy individuals, while a reverse relationship was observed in high self-efficacy cases. In experimental research, received support increased cortisol levels in individuals primed to have high self-efficacy but not those primed to having low efficacy beliefs [29].

To sum up, most research so far has focused either on the effects of self-efficacy or received support on patient or caregiver adaptation to chronic diseases. To the best of our knowledge, only a few studies have investigated the interplay between self-efficacy and support. In addition, researchers rarely based their studies on the dyadic approach [30] in this context, focusing solely on the patients or their caregivers, thus excluding the interdependence factor. Also, since earlier studies were cross-sectional and traditional longitudinal research cases, they investigated only the differences between the participants and time-invariant effects. Therefore, little is known about the possible interactions between the fluctuations in efficacy beliefs and social support for each individual or dyad, and their effects on well-being in disease settings.

### The present study

**Fig 1:**
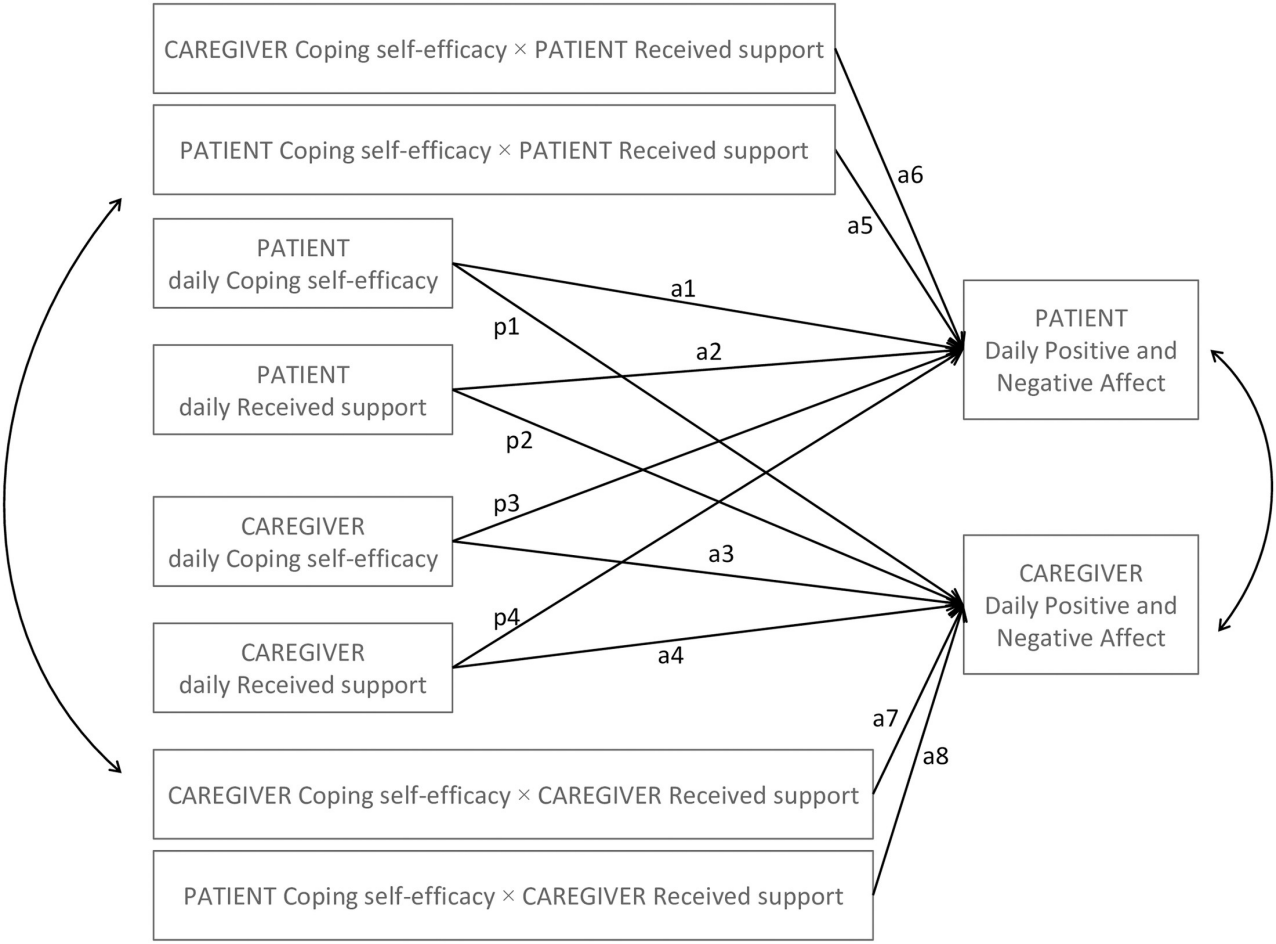
Research model. Note: *paths a5-a8*, interaction effects of coping self-efficacy and received support; *a1*, *a3*, *p1*, *and p3*, main effects of coping self-efficacy; *a2*, *a4*, *p2*, *and p4*, main effects of received support; *a* and *p*, actor and partner effects, respectively; covariances between both, predictors and outcomes are symbolically marked with a two-way arrow.

The present analysis explores the nature of the interaction effect of daily fluctuations in coping self-efficacy and received support on patient and caregiver daily emotional well-being (i.e. positive and negative affect) following intensive cancer treatment (Fig 1). The study is embedded in the dyadic [30] and daily-diary [31] approaches, allowing for the control of dyad interdependence and examination of time-varying effects (i.e. daily fluctuations of the investigated effects or within-dyad effects). It covers dyads during the challenging time (i.e. the first month) following hematopoietic cell transplantation (HCT). This period is preceded by a potentially life-threatening treatment.

HCT is one of the most aggressive but effective forms of treatment for hematological neoplasms [32]. It starts with radio and/or chemotherapy, which destroys the patient hematopoietic system. Next, autologous or allogeneic cell transplantation is performed to restore hematopoietic and immune systems, followed by a period of patient isolation lasting several weeks [32]. During the first month after discharge, the patients experience various side effects of the therapy. Moreover, the patients need to comply with the post-HCT medical regimen, in which caregiver assistance is recommended [33]. HCT affects all domains of patient and caregiver well-being [33, 34], and may require them to cope and activate support. Mutual support and beliefs about the coping ability following HCT may significantly affect their daily emotional well-being.

The present study tested three alternative hypotheses about the effects of daily fluctuations in self-efficacy and received support on daily fluctuations in the affect: higher than typical level of one resource (received support) (a) has a positive effect on daily affect (i.e. compensates for lower self-efficacy days; compensatory effect), or (b) has a negative effect on daily affect (i.e. threatens the affect on high self-efficacy days; interference effect), or (c) higher than typical level of both resources (received support and self-efficacy) has a combined positive effect on daily affect (synergistic effect). Besides the interaction effects, the main effects were also investigated. Based on the social cognitive theory and previous findings, positive main effects of both, daily fluctuations in self-efficacy and received support, were anticipated.

## Materials and methods

### Participants and procedure

Recruitment for the study was conducted in one clinic, approximately two days after admission and before the conditioning treatment. Eligibility criteria for the patients included (*a*) first HCT (autologous or allogeneic), (*b*) age: >18 years, (*c*) no history of other major disabling medical or psychiatric conditions. The caregivers were appointed by the patients. Eligibility criteria for the caregivers included (*a*) close contact and taking care of the patients during the post-HCT outpatient period, (*b*) age: >18 years, and (*c*) no history of major disabling medical or psychiatric conditions. Out of the 561 patients who met the study criteria, 285 patients gave their written informed consent and completed individual background questionnaires (clinical data were obtained from medical records). Among the caregivers, 252 caregivers consented to participate and completed background questionnaires (on the first daily-diary day). Daily assessment took place during the post-HCT outpatient period, which started on day 1 after hospital discharge and lasted 28 consecutive days. All participants were instructed how to complete the diary (particularly with regard to timing and independent diary entries). All dyads completed self-reporting web-based (12.5%) or paper-and-pencil (87.5%) diaries in the evening, each entry took 6–8 minutes. Every evening the participants received a short text message as a reminder. They were also contacted three times during the study period so that the researchers could address any difficulties or emerging questions. Paper versions were returned after the 28-day period. The study protocol was approved by the Ethical Review Board at SWPS University of Social Sciences and Humanities, Faculty of Psychology in Warsaw (Ref. No. 24/2014).

Of the 252 dyads who gave written informed consent, six patients were deemed not eligible for HCT, 17 died during hospitalization, three dyads withdrew their consent, 17 did not return the diaries after the 28-day period, and nine dyads completed fewer than five diary-days. The final sample comprised 200 patient-caregiver dyads (Table 1). Sample attrition analyses indicated that only the type of transplant differentiated the study completers (*N* = 200) and non-completers (*N* = 52). Allogeneic HCT was associated with an increased likelihood of belonging to the non-completers group as compared to autologous HCT; *B* = .98, *SE* = .36, *p* < .001. In the final sample, dyads were mostly married or cohabiting, had at least secondary education, and were in their mid-40s. The patients were mostly male, while the caregivers were mostly female. The majority of the patients were diagnosed with lymphomas and had undergone autologous transplantation.

**Table 1:** Characteristic of patient-caregiver dyads (*N* = 200).

Demographic and clinical variables	Patients	Caregivers
Female (*n*, %)	86 (43)	141 (70.5)
Age (mean, *SD*; years)	47.85 (13.48)	47.38 (13.11)
Education (mean, *SD*; years)	14.18 (3.32)	14.07 (3.29)
Employment: yes (*n*, %)	74 (37.0)	123 (61.5)
Relationship length (mean, *SD*; years)	25.34 (12.26)	25.34 (12.26)
Relationship ties (*n*, %)		
spouse/partner	155 (77.5)	155 (77.5)
mother/father	22 (11.0)	16 (8.0)
daughter/son	16 (8.0)	22 (11.0)
sister/brother	6 (3.0)	6 (3.0)
other	1 (.5)	1 (.5)
Diagnosis (*n*, %)		
leukemias and other myeloid neoplasms	35 (17.5)	--
lymphomas	96 (48.0)	--
multiple myeloma	62 (31.0)	--
other cancer types	7 (3.5)	--
Time since diagnosis (mean, *SD*; months)	21.89 (24.07)	--
Transplant type (*n*, %)		
autologous HCT	148 (74.0)	--
allogeneic HCT	52 (26.0)	--
Isolation length (mean, *SD*; days)	18.51 (9.32)	--
autologous HCT recipients	14.45 (3.52)	--
allogeneic HCT recipients	30.08 (10.91)	--

Leukemias and other myeloid neoplasms include acute lymphoblastic leukemia, acute myeloid leukemia, chronic myelogenous leukemia, myelodysplastic syndrome and myeloproliferative disorders. Lymphomas include Hodgkin and non-Hodgkin type. Other cancer types include solid tumors and other cancers.

### Daily diary measures

#### Coping self-efficacy

The participants completed six items from the Coping Self-efficacy Scale (CSE) [35] adapted to the daily procedure. They rated the extent of confidence in performing coping behaviors with own/partner’s health problems (e.g. “Made a plan of action and followed it when confronted with my/partner’s health problems”, “Made unpleasant thoughts go away”) on a given day using a 5-point scale, ranging from 1 (“not at all”) to 5 (“strongly”). Higher scores indicated higher daily coping self-efficacy (total daily score: 6–30; between-dyad means were 20.41±4.89 and 19.20±5.24 for the patients and the caregivers, respectively). Within-dyad reliabilities (omega coefficients) [36] were .87 for both study partners, while between-dyad reliabilities were .90 and .86 for the patients and the caregivers, respectively.

#### Received social support

The participants completed six items from the Berlin Social Support Scale (BSSS) [37] adapted to the daily procedure. They rated the extent of emotional, informational, and instrumental support received from the study partner (e.g. “They listened to me and showed understanding of my feelings”) on a given day using a 4-point scale, ranging from 1 (“not at all”) to 4 (“very strongly”). Higher scores indicated higher daily received support (total daily score: 6–24; between-dyad means were 18.20±4.22 and 16.37±4.70 in the patients and the caregivers, respectively). Within-dyad reliabilities were .89 for both study partners, while between-dyad reliabilities were .90 and .91 for the patients and the caregivers, respectively.

#### Positive and negative affect

The participants rated how they felt on a given day using a 7-point scale ranging from 1 (“not at all”) to 7 (“very strongly”). They assessed six positive affect items (happy, enthusiastic, content, pleased, excited, relaxed) and six negative affect items (unhappy, irritable, bored, sad, nervous, sluggish). Based on the Circumplex Model of Emotion by Larsen and Diener [38], the adjectives reflected neutral, low, and high intensity. Higher scores indicated higher daily positive or negative affect (total daily score per scale: 6–42; between-dyad means for positive and negative affect were 14.75±4.72 and 9.72±3.50, respectively in the patients, and 15.52±4.57 and 9.64±3.79, respectively in the caregivers). Within-dyad reliabilities were .89 for both study partners, whereas between-dyad reliabilities ranged from .90 to .94.

### Statistical analysis and data preparation

Multilevel structural equation modeling (MSEM) was used to explore the interaction effect of daily fluctuations in coping self-efficacy and received support in patient-caregiver dyads. MSEM estimated within-dyad fluctuations (i.e. daily deviations from the personal mean for each dyad member) and between-dyad differences (i.e. differences in means across all participants and days) simultaneously. The present study focused on the within-dyad fluctuations only. The analysis was based on the actor-partner interdependence moderation model [39] and was conducted using the Mplus statistical package version 8 [40]. Modified code provided by Laurenceau and Bolger [41] was applied. To separate the correlation effects from the short-time predictions, the concurrent (i.e. same-day) and lagged (i.e. next-day) effects were tested. In the concurrent analysis, daily positive and negative affect (for both, patients and caregivers) were predicted by person-centered (i.e. the deviation from the personal mean for each participant) daily coping self-efficacy (Fig 1, paths a1, a3, p1, p3), received support (paths a2, a4, p2, p4), coping self-efficacy by received support interaction (paths a5-a8), and the linear time trend (centered on the middle time point). In detail, we estimated random effects for pairs of intercepts (of positive and negative affect, simultaneously), actor and partner effects (of coping self-efficacy and received support; paths a1-a4 and p1-p4, respectively), and four interaction effects (pairs of actor support by actor coping-self efficacy and actor support by partner self-efficacy; paths a5-a8) for both, the patients and the caregivers [39]. Actor effects refer to the effects within a person (i.e. patient or caregiver), while partner effects refer to the effects across dyad members (i.e. from patient to caregiver and vice versa) [30]. In the lagged analysis, previous-day affect was controlled next to previous-day predictors, which allowed to predict the affect change. In both analyses, possible covariates were controlled (i.e. age, sex, education, employment, relationship ties/length, and transplant type). All these variables were significantly related to the participant affect in the preliminary analysis. Significant interaction effects were probed using the Johnson-Neyman technique, which identifies the regions of significance for the interaction [42]. The maximum likelihood was used as an estimator.

One hundred and forty-one dyads (70.5%) completed at least 26 diary-days. Mean number of diary-days was 26.21±4.47 and 25.68±4.45 for the patients and the caregivers, respectively. Missing data were below 11% (ranging from 7.1% for patient daily coping self-efficacy to 10.8% for caregiver received support). The final dataset consisted of 4,674 daily reports from 200 dyads. Missing data analysis revealed no significant associations between missing records and the analyzed variables (i.e. daily coping self-efficacy, received support, and positive and negative affect in both, patients and caregivers). Missing data were handled during model estimation using a full information maximum likelihood method, which is the most recommended tool for missing data management [43].

## Results

**Fig 2:**
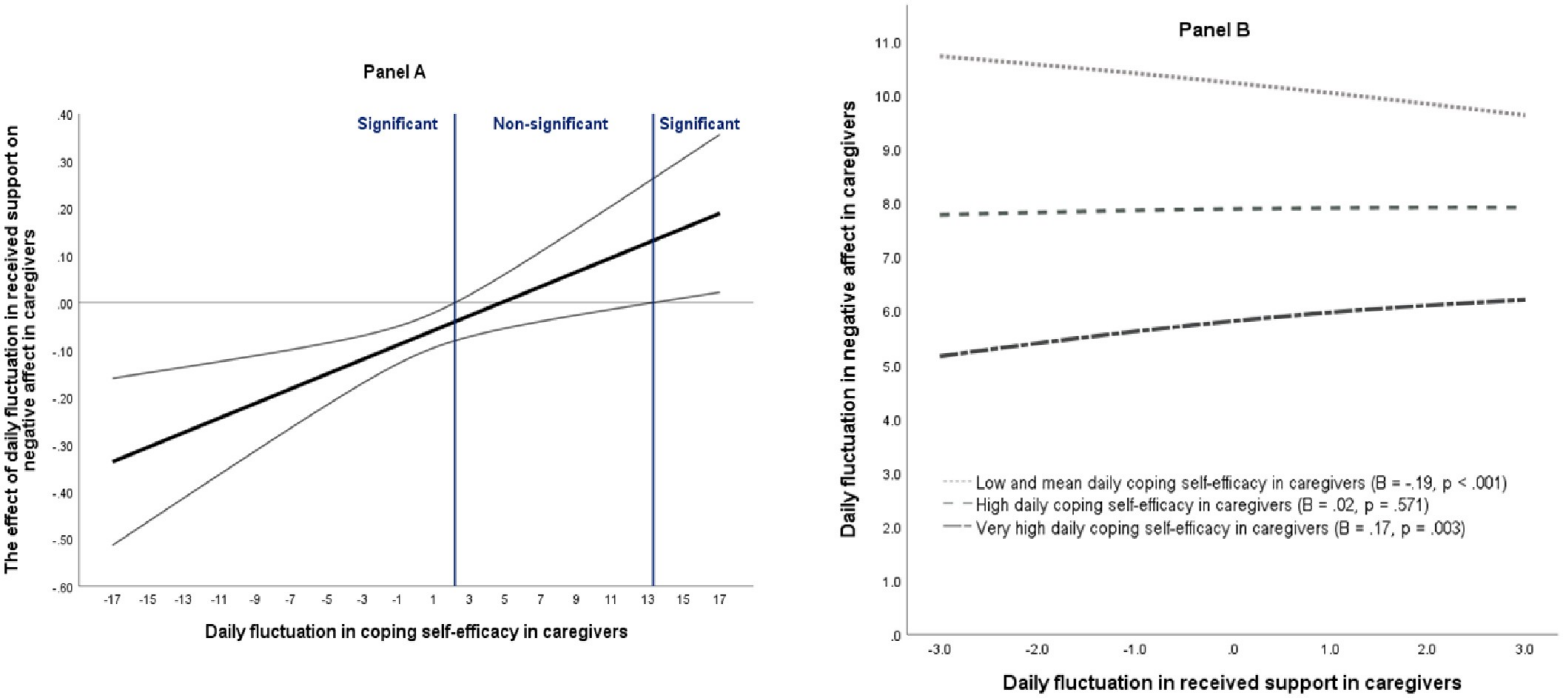
Daily received support by self-efficacy effect on concurrent negative affect in the caregivers. Panel A = The regions of significance of interaction (solid line = interaction effect, thin lines = 95% confidence interval; when the y-zero-line is included in the confidence bands, the effect of daily deviations in received support on negative affect is not significant). Panel B = Simple slopes for the days with typical and lower than typical (below 2.17 on a scale from -17 to 17), higher than typical, and much higher than typical (above 13.28) daily self-efficacy.

The results of the concurrent multilevel structural equation modeling are shown in Table 2. The effect of resource interaction on the fluctuations in caregiver negative affect was statistically significant (path a7, Fig 1), supporting the interference of resources in the caregivers. Fig 2 depicts the concurrent association between caregiver daily received support and negative affect as a function of daily coping self-efficacy. On days when coping self-efficacy was typical or lower than typical (i.e. lower than on an average day), higher received support was associated with lower negative affect in the caregivers. An opposite association was observed when the level of coping self-efficacy was much higher than typical. Contrary to expectations, daily fluctuations in coping self-efficacy did not contribute significantly to the effect of received support on affect, either in the patients or the partners.

**Table 2:** Results of concurrent analysis: The fixed effects of daily coping self-efficacy (CSE), received support (RS), and their interaction on same-day affect in 200 patient-caregiver dyads.

Outcome:	Patient				Caregiver			
	Positive affect		Negative affect		Positive affect		Negative affect	
Predictor	*Est*.*(SE)*	95% *CI*	*Est*. *(SE)*	95% *CI*	*Est*. *(SE)*	95% *CI*	*Est*. *(SE)*	95% *CI*
Intercept	14.79 (.26) ***	[14.37; 15.21]	9.75 (.19) ***	[9.43; 10.06]	15.59 (.24) ***	[15.20; 15.98]	9.42 (.20) ***	[9.09; 9.76]
Patient CSE	.33 (.03)***	[.27; .38]	-.19 (.03)***	[-.24; -.14]	.07 (.02)**	[.04; .11]	-.04 (.02)*	[-.07; -.01]
Patient RS	.03 (.03)	[-.02; .07]	.04 (.02)	[-.01; .07]	-.05 (.02)*	[-.09; -.01]	.01 (.02)	[-.03; .04]
Patient RS × CSE	-.01 (.01)	[-.02; .01]	.01 (.01)	[-.01; .02]	-	-	-	-
Patient RS × Caregiver CSE	.00 (.02)	[-.04; .04]	.00 (.01)	[-.02; .02]	-	-	-	-
Caregiver CSE	.03 (.02)	[-.01; .07]	-.01 (.02)	[-.04; .02]	.28 (.03)***	[.23; .33]	-.19 (.02)***	[-.23; -.15]
Caregiver RS	.07 (.02)***	[.04; .10]	-.07 (.02)***	[-.10; -.04]	.11 (.02)***	[.08; .15]	-.07 (.02)***	[-.10; -.04]
Caregiver RS × CSE	-	-	-	-	.00 (.01)	[-.01; .01]	.02 (.01)**	[.01; .03]
Caregiver RS × Patient CSE	-	-	-	-	.00 (.01)	[-.01; .01]	.01 (.01)	[-.01; .02]
***Random effects***								
Intercept variance	10.35 (1.19)	[8.39; 12.32]	5.66 (.47)	[4.89; 6.43]	8.98 (1.01)	[7.32; 10.64]	7.58 (.95)	[6.02; 9.14]
Residual variance	5.74 (.35)	[5.16; 6.33]	3.96 (.31)	[3.46; 4.47]	7.31 (.49)	[6.50; 8.12]	4.93 (.35)	[4.35; 5.50]

Confounders (i.e. age, sex, education, employment, relationship ties/length, and transplant type) were controlled for in the analysis.

Between-person effects are provided in S1 Table.

*p < .05;

**p < .01;

***p < .001.

As far the main actor and partner effects are concerned, in line with earlier predictions, higher than typical daily coping self-efficacy and received support were significantly associated with higher daily positive affect and lower negative affect in the caregivers (paths a3 and a4, respectively). In the patients, only daily fluctuations in coping self-efficacy contributed significantly to their better concurrent affect, both positive and negative (path a1). In addition, patient-reported daily fluctuations in coping self-efficacy were predictive of higher same-day positive affect and lower negative affect in the caregivers (path p1). The abovementioned associations were not observed in the patients. Instead, caregiver-reported daily fluctuations in received support were associated with better concurrent affect in the patients, both positive and negative (path p4). Patient-reported higher than typical daily received support contributed to lower positive affect in the caregivers.

In the lagged analysis (Table 3), only the main actor effect of daily fluctuations in coping self-efficacy on positive affect in the patients was statistically significant (path a1). Higher than typical daily coping self-efficacy increased next-day positive affect in the patients. The remaining main and interaction effects did not contribute significantly to the fluctuation of daily positive and negative affect in either party.

**Table 3:** Results of lagged analysis: The fixed effects of daily coping self-efficacy (CSE), received support (RS), and their interaction on next-day affect in 200 patient-caregiver dyads.

Outcome:	Patient				Caregiver			
	Next-day Positive affect		Next-day Negative affect		Next-day Positive affect		Next-day Negative affect	
Predictor	*Est*.*(SE)*	95% *CI*	*Est*. *(SE)*	95% *CI*	*Est*. *(SE)*	95% *CI*	*Est*. *(SE)*	95% *CI*
Intercept	14.75(.26)***	[14.33; 15.18]	9.70(.19)***	[9.38; 10.01]	15.60 (.50)***	[14.78; 16.41]	9.47 (.30)***	[8.98; 9.95]
Patient CSE	.08(.02)***	[.04; .11]	-.01(.02)	[-.05; .03]	.01(.05)	[-.06; .08]	.01(.02)	[-.03; .05]
Patient RS	.01(.07)	[-.11; .13]	.02(.02)	[-.01; .05]	-.03(.04)	[-.09; .04]	-.01(.02)	[-.04; .03]
Patient RS × CSE	.00(.04)	[-.05; .06]	.00(.02)	[-.04; .04]	-	-	-	-
Patient RS × Caregiver CSE	.00(.03)	[-.05; .05]	.00(.01)	[-.01; .01]	-	-	-	-
Caregiver CSE	.00(.03)	[-.05; .05]	-.02(.02)	[-.05; .01]	.07(.08)	[-.06; .19]	-.01(.03)	[-.06; .04]
Caregiver RS	.03(.05)	[-.05; .11]	-.02(.02)	[-.05; .01]	.05(.06)	[-.05; .15]	-.04(.02)	[-.08; .00]
Caregiver RS × CSE	-	-	-	-	.02(.02)	[-.02; .03]	.00(.01)	[-.01; .02]
Caregiver RS × Patient CSE	-	-	-	-	.01(.05)	[-.07; .09]	.00(.03)	[-.05; .04]
***Random effects***								
Intercept variance	10.13(1.3)***	[7.97; 12.29]	5.64(.69)***	[4.50; 6.78]	9.11(.93)***	[7.58; 10.64]	7.52(1.24)***	[5.48; 9.56]
Residual variance	5.94(.47)***	[5.16; 6.72]	4.03(.34)***	[3.48; 4.58]	8.04(.79)***	[6.75; 9.33]	5.18(.36)***	[4.59; 5.77]

Previous day affect and confounders (i.e. age, sex, education, employment, relationship ties/length, and transplant type) were controlled for in the analysis. Between-person coefficients are presented in S1 Table.

***p < .001.

## Discussion

This study aimed to address the gap in our understanding of the interaction effect of self-efficacy beliefs and received support in dyads facing cancer treatment. To the best of our knowledge, this has been the first attempt to investigate daily fluctuations in coping self-efficacy, received support, and their interaction effect on patient-caregiver affect following HCT. The findings demonstrate beneficial effect of days with higher than typical coping self-efficacy and received support in both, patients and caregivers, as well as an interference rather than synergistic or compensatory effect of those resources in the affected dyads.

### Interference effect of the resources in the caregivers

The interplay between daily fluctuations in coping self-efficacy and received support contributed significantly to caregiver fluctuations in daily negative affect only, suggesting the interference interaction effect of the resources. Higher daily received support was associated with higher caregiver negative affect on days when self-efficacy was much higher than typical, indicating optimal daily received support on typical or lower self-efficacy days. In comparison, earlier studies found the interference effect in patient populations [28, 29]. It is possible that on days when self-efficacy was significantly increased, social support was not expected and it undermined the sense of competence in the caregivers, harming their self-esteem. Resource needs were competing or conflicting [25]. During the first month after HCT, the caregivers are faced with several patient-related challenges (special dietary needs, medicine administration, hygiene, etc.) and other tasks (e.g. childcare, professional work, chores, etc.). Feelings of incompetence or shortcomings may have affected the level of negative affect. Due to the fact that other resources can contribute to resource conflict as well, additional research is needed to test possible confounders or other moderators, such as support need or effectiveness. Further research to differentiate types of received support is also necessary.

Alternatively—since negative effect was detected on days with fluctuation towards much higher than typical efficacy beliefs and the beliefs were declarative—extremely high self-efficacy could have been illusory and did not translate into the use of high received support. Caregiver burden may have resulted in strong feelings of pressure, also to manage external and internal demands. Extremely high daily pressure and self-efficacy could have coexisted and jointly limited the possibility to use support. By comparison, prior longitudinal research in cancer patients indicated that perceived self-efficacy may elicit received support, resulting in better outcomes [44]. Similar research about the caregivers has been scarce. Our findings may bridge the gap in the knowledge about the nature of the interplay between daily fluctuations in self-efficacy beliefs and received support, although further research is necessary to investigate the mechanism of temporal associations in daily fluctuations of resource compounds within patient-caregiver dyads.

Interestingly, the interference effect emerged in negative affect and same-day analysis only. Most likely, this is due to the distinct mechanisms of positive and negative affect elicitation [45]. Fluctuations in the positive affect were associated with independent operation of daily efficacy beliefs and received support in the caregivers. The cross-sectional nature of the dependencies does not exclude the inverse relationship between the investigated variables, where affect determines the assessment of daily coping self-efficacy and received support.

### Beneficial effect of patient daily coping self-efficacy

When analyzing the effect of the resources separately, higher than typical daily coping self-efficacy was significantly associated with better same-day daily affect in both, patients and caregivers, while beneficial effects of higher daily received support were observed in caregivers only. The obtained results are consistent with the social cognitive theory [3] and earlier cross-sectional or longitudinal research in post-HCT patients [10, 11], patients with various cancer types [5–9, 12, 14, 16, 18–21, 46], and their caregivers [12, 13, 19, 21]. In patients, the effect of daily fluctuations in received support was null, thus supporting earlier findings in cancer contexts [22, 23] and in non-clinical populations [47, 48], whereas daily coping self-efficacy played a significant, positive role in the fluctuations of patient daily affect. The benefits were especially related to positive affect, for which the effect persisted on the following day. Possibly, on days when coping-self efficacy was higher than typical, the patients experienced higher perceived controllability of external and internal demands, perceived the demands as less stressful, and chose adequate coping strategies [3, 24, 35]. As our patients were rather limited by various medical regimens and their own physical condition, they may have felt dependent on support receipt. Daily coping self-efficacy beliefs may have abolished those limitations. However, additional research is necessary to address the indirect mechanisms of daily fluctuations between self-efficacy and affect. Our earlier findings demonstrated that fluctuations in daily coping self-efficacy modified the association between daily coping strategies and affect [49].

In our study, higher daily coping self-efficacy in the patients also contributed to better same-day affect in the caregivers. Similar results, although not related to within-dyad effects, were found in prostate cancer patients and their caregivers [12]. A possible explanation may also be linked to patient self-confidence and independence as well as more effective coping [3, 24, 35], which was transferred to the caregiver. Alternatively, caregiver satisfaction could be transferred to the patient, reinforcing their belief in coping self-efficacy. Cross-partner effects also emerged in relation to daily fluctuations in received support, although they were less consistent. Indeed, our findings highlight the complexity of mutual relations in a close relationship and how a particular resource may be good or neutral to one party but less beneficial to the other. It is possible that demand for support, received support quality, and the quality of the patient-caregiver relationship would allow for an in-depth data interpretation.

### Practical implications

The results of our study could be encouraging for dyadic intervention research in HCT recovery. Firstly, daily fluctuations in coping self-efficacy and received support may be associated with affect fluctuations in cancer populations. This finding expands earlier cross-sectional and longitudinal research on self-efficacy beliefs and support receipt in disease contexts. Secondly, our findings suggest that enhancement of coping self-efficacy beliefs in patients and their caregivers may by beneficial for both parties and foster their daily well-being. Then, high coping self-efficacy beliefs could be launched on a specific day or occasion. Previous intervention studies demonstrated the usefulness of self-efficacy enhancement programs in cancer patients and their caregivers [50–52]. However, further studies are necessary to investigate the benefits of dyadic interventions in this area. Finally, the effect of daily coping self-efficacy can be enhanced in the caregivers by providing them with optimal daily support. To date, the effectiveness of combined intervention has been partially tested in men with prostate cancer only [53]. Further intervention studies are necessary to elucidate the effectiveness of combined interventions in the caregivers. As medical care is predominantly patient-centered, there is a distinct need for support venues or web-based interventions for caregivers, which would combine evidence-based practices aimed at optimal augmentation of both resources.

### Study limitations

The generalizability of the study results to all cancer patients and their caregivers is limited due to the specificity of the HCT procedure. The experience of HCT and its consequences goes beyond the experience of patients treated with standard chemo-/radiotherapy, post-surgery, newly diagnosed or cancer survivors, i.e. the focus groups of most studies. So, further daily-dyadic studies on the interplay between individual and social resources in other cancer populations are needed. Secondly, women were overrepresented among the caregivers. Despite controlling for gender, it is possible that the effects in the caregivers are more representative of women. Thirdly, three-quarters of the patients underwent autologous HCT, which is characterized by shorter hospitalization and separation from the family as compared to allogeneic HCT. That may have affected the study findings, despite controlling for transplant type. Next, self-reports were collected at a relatively high frequency (28 consecutive days), which could have biased the results (subjectivity, learning the test). Also, most dyads completed paper diaries whose filling time is more difficult to monitor than electronic ones. Finally, the analysis was limited to investigating interaction effect of day-by-day fluctuation in coping self-efficacy beliefs and received support. Future research is needed to test the possible indirect paths in the associations between daily fluctuations in coping self-efficacy, received support, and affect within patient-caregiver dyads.

## Conclusions

Our study has been the first report of the effect of the interplay between daily fluctuations in coping self-efficacy beliefs and received support in patient-caregiver dyads undergoing a demanding cancer treatment. In the caregivers, daily received support varied in its positive and negative role, which depended on daily fluctuations in efficacy beliefs, whereas the patients benefited from high daily self-efficacy.

## Supporting information

S1 Table Results of between-dyad level effects [Estimate (Est.) and Standard Error (SE)] of coping self-efficacy (CSE), received support (RS), and their interaction on affect in 200 patient-caregiver dyads (in the same- and next-day analyses). Legend: PA, positive affect, NA, negative affect. *p < .05; **p < .01; ***p < .001.
(PDF)
