# Supplementary material for: Interaction effect of coping self-efficacy and received support in daily life of hematopoietic cell transplant patient-caregiver dyads
Source: PLoS One. 2021 Nov 17;16(11):e0260128. doi: 10.1371/journal.pone.0260128 (PMC8598009; doi:10.1371/journal.pone.0260128)
Supplement: S1 Table — Legend: PA, positive affect, NA, negative affect. *p < .05; **p < .01; ***p < .001. (PDF) [file pone.0260128.s001.pdf]

Supplementary Materials

Interaction effect of coping self-efficacy and received support in daily life of hematopoietic cell transplant patient-caregiver dyads

Table S1. Results of between-dyad level effects [Estimate (Est.) and Standard Error (SE)] of coping self-efficacy (CSE), received support (RS), and their interaction on affect in 200 patient-caregiver dyads (in the same- and next-day analyses).

Outcome	**Same-day analysis *[Est.(SE)]***				**Next-day analysis *[Est.(SE)]***			
	**Patient**		**Caregiver**		**Patient**		**Caregiver**	
**Predictor**	**PA**	**NA**	**PA**	**NA**	**PA**	**NA**	**PA**	**NA**
Patient CSE	.48 (.08)***	-.14 (.05)**	.07(.06)	-.03 (.05)	.47 (.11)***	-.11 (.05)*	.09 (.09)	-.02 (.05)
Patient RS	-.06 (.07)	.17 (.06)**	-.16 (.07)**	.06 (.07)	-.05 (.08)	.18 (.06)**	-.16 (.08)*	.07 (.08)
Patient RS × CSE	.03 (.02)	-.02 (.01)	-	-	.02 (.03)	-.01 (.01)	-	-
Patient RS × Caregiver CSE	-.04 (.02)*	.02 (.01)	-	-	-.04 (.02)*	.02 (.02)	-	-
Caregiver CSE	-.06 (.07)	-.05 (.05)	.16 (.06)**	-.11 (.05)*	-.07 (.12)	-.08 (.05)	.20 (.21)	-.11 (.07)
Caregiver RS	.11 (.08)	-.06 (.06)	.33 (.07)***	-.05 (.06)	.12 (.21)	-.05 (.06)	.31 (.10)**	-.05 (.09)
Caregiver RS × CSE	-	-	.01 (.01)	.02 (.01)	-	-	.00 (.06)	.02 (.02)
Caregiver RS × Patient CSE	-	-	.00 (.01)	.00 (.01)	-	-	.00 (.02)	.00 (.02)

PA, positive affect, NA, negative affect.

*p < .05; **p < .01; ***p < .001.
